# Supplementary material for: Alkaloid Concentrations of Lolium perenne Infected with Epichloë festucae var. lolii with Different Detection Methods—A Re-Evaluation of Intoxication Risk in Germany?
Source: J Fungi (Basel). 2020 Sep 18;6(3):177. doi: 10.3390/jof6030177 (PMC7558822; doi:10.3390/jof6030177)
Supplement: Supplementary file 1 [file jof-06-00177-s001.pdf]

Supplemental material

Figure S1: Chromatograms of analytes analyzed with different methods: **A:** Chromatograms of peramine, homoperamine, LSD-d3, ergovaline and ergotamine analyzed with UPLC-MS/MS method 1 from [1], reconstituted in water/acetonitrile/formic acid 80:20:0.1 (v/v/v), **B:** Chromatograms of peramine, homoperamine, LSD-d3, ergovaline, ergotamine, paxiline and lolitrem B analyzed with UPLC-MS/MS method 1 [1], reconstituted in water/acetonitrile/formic acid 50:50:0.1 (v/v/v), **C:** Chromatograms of peramine, homoperamine, ergovaline, ergotamine and lolitrem B analyzed with UPLC-MS/MS method 2 from [2], reconstituted in methanol (80%).

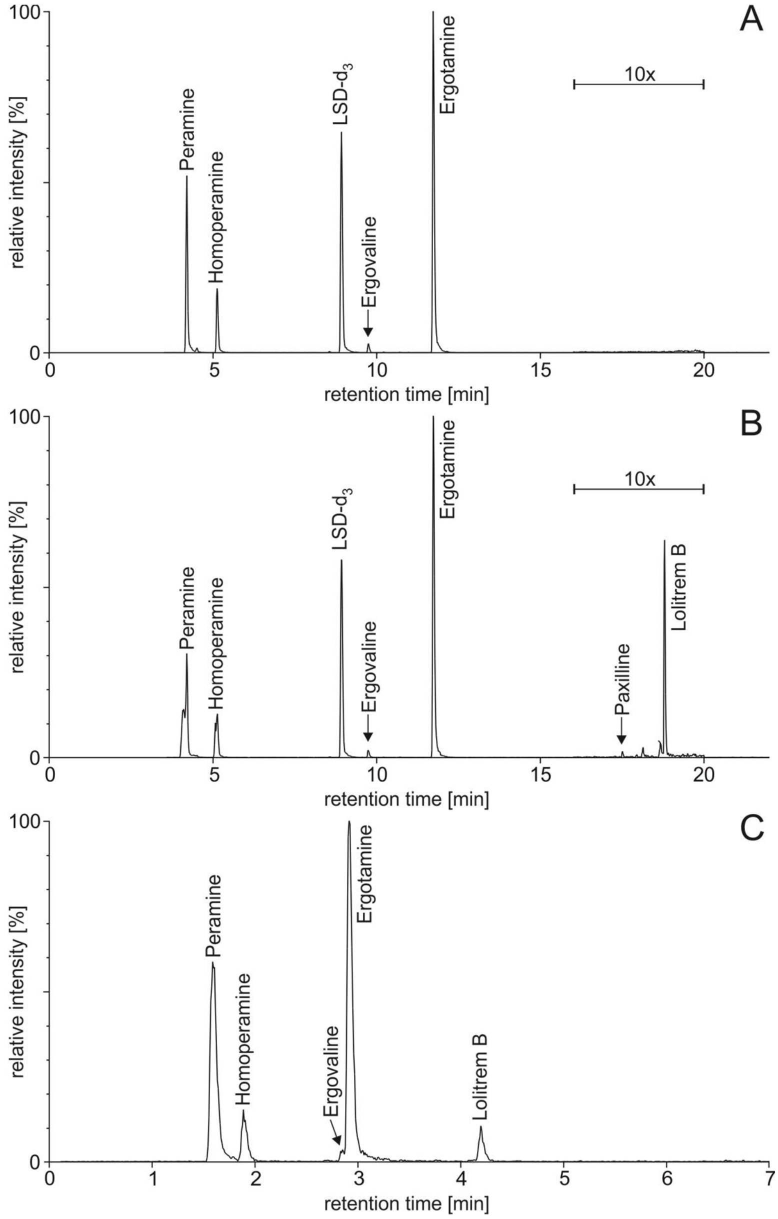

Table S1: Study sites with alkaloid concentrations above toxicity thresholds in 2015: Number of samples with concentrations above toxicity thresholds in 2015 for peramine, lolitrem B and ergovaline and number of infected samples at the study site. Sampled in summer (July/August) 2015.

**Study site**	**No. of samples with Peramine concentrations >2 µg/g**	**No. of samples with Lolitrem B concentrations >1.8 µg/g**	**No of samples with Ergovaline concentrations >0.3 µg/g**	**No of infected samples/total sample size**
SEG40	2	0	1	2/20
SEG43	1	5	0	5/20
SEG44	2	3	0	3/20
SEG46	6	9	0	13/20
SEG47	2	4	0	8/20

Table S2: Differences between UPLC-methods used in dataset (3)

Name of the method	Method 1	Method 2
Publications	[1]<br>They used dry plant weight	[2,3]<br>They used fresh plant weight
Detectable alkaloids	Lolitrem B, ergovaline, peramine, paxilline	Lolitrem B, ergovaline, peramine
Standards	Homoperamine, ergotamine, LSD-d3	Homoperamine, ergotamine
Substances for validation	Peramine, lolitrem B, ergovaline, paxilline	Peramine, lolitrem B
extraction	One part of the sample was reconstituted in water/acetonitrile/formic acid 50:50:0.1 (v/v/v) for the analysis of lolitrem B and paxilline<br>the other in water/acetonitrile/formic acid 80:20:0.1 (v/v/v) for the analysis of peramine and ergovaline	Samples were reconstituted in methanol (80 %)
Analytical method	UPLC-MS/MS	UPLC-MS/MS
Column	Aquitiy UPLC column (100x2.1 mm; 1.7 µm; Waters GmbH, Eschborn, Germany), Reverse Phase	Acquitiy UPLC BEH column (50x2.1 mm; 1.7 µm; Waters GmbH, Eschborn, Germany), Reverse Phase
solvents	0.1 % formic acid dissolved in water (solvent A) and acetonitrile containing 0.1 % formic acid (solvent B)	0.1 % formic acid dissolved in water (solvent A) and acetonitrile containing 0.1 % formic acid (solvent B)

Flow rate	0.4 ml/min	0.3 ml/min
gradient	0–1.0 min 98% solvent A, 1.0–3.0 min to 90% solvent A, 3.0–5.0 min to 85% solvent A, 5.0–7.5 min to 80% solvent A, 7.5–10.0 min to 75% solvent A, 10.0–11.5 min to 70% solvent A, 11.5–13.0 min to 65% solvent A, 13.0–14.5 min to 50% solvent A, 14.5–16.0 min to solvent 40% A, 16.0–19.0 min to 0% solvent A, 19.0–22.0 hold 0% solvent A, 22.0–23.0 back to 98% solvent A and hold for another 2 min	from 5% to 25% solvent B in 5 min, followed by 25% to 75% solvent B in 0.5 min, then 75% to 100 % solvent B in 2.5 min
Total run time	25 min	10 min
Injection volume	10 µl for the analyses of peramine and ergovaline<br>5 µl for the analyses of lolitrem B and paxilline.	5 µl
Limit of detection	Limit of detection (LOD): Paxilline, lolitrem B: 0.05 ng on column<br>Ergovaline, peramine: 0.01 ng on column.	LOD: comparable to method 1

Table S3: Mean alkaloid concentrations (in [µg/g] dry weight (mean ± SE)) per month on the five study sites in the field in 2018.

**Month**	**Year**	**Peramine**	**Lolitrem B**
April	2018	0.032 ± 0.018	0.000 ± 0.000
June	2018	0.773 ± 0.158	0.601 ± 0.113
July	2018	0.939 ± 0.233	0.367 ± 0.243
September	2018	3.227 ± 0.611	0.665 ± 0.164
November	2018	0.345 ± 0.110	0.010 ± 0.008
January	2019	0.242 ± 0.123	0.001 ± 0.001

Table S4: Mean alkaloid concentrations based on plant fresh or dry weight from April until September 2019 in the common garden experiment (mean ± SE, n=10 per time point). Date: dd.mm.yyyy, Week: calendar week. NA: not measured, nd: not detected

**sample**	**Date**	**Week**	**Peramine**		**Ergovaline**		**Lolitrem B**		**Paxilline**	
			**fresh**	**dry**	**fresh**	**dry**	**fresh**	**dry**	**fresh**	**dry**
1	09. April 2019	15	NA	26.21 ± 2.97	NA	0.40 ± 0.14	NA	0.29 ± 0.07	NA	0.10 ± 0.06
2	24. April 2019	17	NA	16.42 ± 1.71	NA	0.32 ± 0.12	NA	0.14 ± 0.07	NA	0.20 ± 0.10
3	07. May 2019	19	3.02 ± 0.29	8.44 ± 1.13	0.11 ± 0.03	0.48 ± 0.13	0.59 ± 0.12	1.46 ± 0.37	nd	nd
4	21. May 2019	21	NA	1.02 ± 0.10	NA	0.03 ± 0.01	NA	0.03 ± 0.004	NA	nd
5	04. June 2019	23	2.69 ± 0.31	9.29 ± 0.99	0.13 ± 0.05	0.49 ± 0.14	nd	0.05 ± 0.02	nd	nd
6	18. June 2019	25	4.21 ± 0.47	17.28 ± 2.33	0.26 ± 0.06	1.33 ± 0.30	0.01 ± 0.01	0.10 ± 0.04	nd	nd
7	02. July 2019	27	4.05 ± 0.60	13.24 ± 2.16	0.18 ± 0.04	0.65 ± 0.16	0.34 ± 0.04	1.28 ± 0.17	nd	nd
8	16. July 2019	29	7.57 ± 0.92	22.06 ± 2.76	0.34 ± 0.06	1.30 ± 0.26	0.70 ± 0.13	2.00 ± 0.32	nd	nd
9	30. July 2019	31	10.78 ± 0.700	31.81 ± 3.08	0.31 ± 0.04	1.03 ± 0.17	nd	0.03 ± 0.03	nd	0.05 ± 0.03
10	14. August 2019	33	13.46 ± 1.26	35.63 ± 3.10	0.43 ± 0.10	1.37 ± 0.28	2.51 ± 0.33	6.57 ± 1.09	nd	0.13 ± 0.07
11	28. August 2019	35	8.21 ± 0.98	28.49 ± 2.80	0.39 ± 0.08	1.43 ± 0.24	0.27 ± 0.07	0.71 ± 0.14	nd	0.32 ± 0.13
12	10. September 2019	37	8.58 ± 0.64	22.76 ± 1.94	0.51 ± 0.08	1.43 ± 0.18	0.63 ± 0.07	nd	0.02 ± 0.02	0.56 ± 0.28
13	25. September 2019	39	5.56 ± 1.17	16.32 ± 2.30	0.39 ± 0.09	0.81 ± 0.18	0.03 ± 0.03	nd	nd	0.07 ± 0.04

1. Krauss, J.; Vikuk, V.; Young, C.A.; Krischke, M.; Mueller, M.J.; Baerenfaller, K. *Epichloë* endophyte infection rates and alkaloid content in commercially available grass

seed mixtures in Europe. *Microorganisms* **2020**, *8*, 498, doi:10.3390/microorganisms8040498.
2. Fuchs, B.; Krischke, M.; Mueller, M.J.; Krauss, J. Peramine and lolitrem B from endophyte-grass associations cascade up the food chain. *Journal of Chemical Ecology* **2013**, *39*, 1385–1389, doi:10.1007/s10886-013-0364-2.
3. König, J.; Fuchs, B.; Krischke, M.; Mueller, M.J.; Krauss, J. Hide and seek - Infection rates and alkaloid concentrations of *Epichlo*ë *festucae* var. *lolii* in *Lolium perenne* along a land-use gradient in Germany. *Grass and Forage Science* **2018**, *73*, 510–516, doi:http://doi.org/10.1111/gfs.12330.
